# Adipose Tissue- and Bone Marrow-Derived Mesenchymal Stem Cells from Sheep: Culture Characteristics

**DOI:** 10.3390/ani11082153

**Published:** 2021-07-21

**Authors:** Ejaz R. Dar, Mudasir B. Gugjoo, Moien Javaid, Shahid Hussain, Mujeeb R. Fazili, Kuldeep Dhama, Taha Alqahtani, Ali M. Alqahtani, Riaz A. Shah, Talha Bin Emran

**Affiliations:** 1 Division of Surgery and Radiology, Faculty of Veterinary Sciences and Animal Husbandry, SKUAST, Shuhama, Srinagar 190006, India; drejaz5106@gmail.com; 2 Division of Veterinary Clinical Complex, Faculty of Veterinary Sciences and Animal Husbandry, SKUAST, Shuhama, Srinagar 190006, India; moinjavaid@gmail.com (M.J.); shahidhussainsofi@live.com (S.H.); fazili_mr@yahoo.co.in (M.R.F.); 3 Division of Pathology, ICAR-Indian Veterinary Research Institute, Izatnagar, Bareilly 243122, India; kdhama@rediffmail.com; 4 Department of Pharmacology, College of Pharmacy, King Khalid University, Abha 62529, Saudi Arabia; ttaha@kku.edu.sa (T.A.); amsfr@kku.edu.sa (A.M.A.); 5 Division of Animal Biotechnology, Faculty of Veterinary Sciences and Animal Husbandry, SKUAST, Shuhama, Srinagar 190006, India; drriazshah@gmail.com; 6 Department of Pharmacy, BGC Trust University Bangladesh, Chittagong 4381, Bangladesh

**Keywords:** adipose tissue, bone marrow, cell proliferation, differentiation, mesenchymal stem cell, sheep

## Abstract

**Simple Summary:**

Mesenchymal stem cells (MSCs) are available in minuscule numbers in the body or placental tissues. These cells have mostly been harvested from bone marrow and adipose tissue. To broaden the currently available knowledge, the current study provides (a) information on the feasibility of isolation of MSCs at different ambient temperatures, (b) details of MSCs’ culture characteristics with respect to the physiological status of the donor, and (c) information on the viability of cryopreserved cells. Bone marrow harbors a higher mononuclear cell fraction than that of the adipose tissue, although percent adherent cells are comparably more in adipose tissue. MSCs from a pregnant donor show enhanced proliferation and differentiation potential, although further studies are desired. The cryopreserved cells have comparable characteristics to that of the fresh cells. In conclusion, donor animals’ tissue type and physiological status may affect MSCs’ characteristics and should be taken into consideration while applying in clinical settings.

**Abstract:**

The current study demonstrates the culture characteristics of adipose tissue and bone marrow-derived mesenchymal stem cells (MSC). The study evaluates the effect of ambient temperature, physiological status of the donor and the tissue source on sheep (*Ovis aries*) mesenchymal stem cells. The tissue samples were harvested from full term pregnant female sheep (*n* = 9) and male sheep (*n* = 10). Adipose tissue was harvested from *n* = 9 sheep and bone marrow from *n* = 10 sheep. The samples (adipose tissue, *n* = 2; bone marrow, *n* = 3) transported at cold ambient temperature (<10 °C) failed to yield MSCs while those (*n* = 14) at higher (>20 °C) ambient temperature successfully yielded MSCs. Bone marrow mononuclear cell (MNC) fraction was higher than the adipose tissue-derived stromal vascular fraction (SVF), but the percent adherent cells (PAC) was higher in the later cell fraction. Adipose tissue-derived MSCs from the full term female sheep had a significantly (*p* < 0.05) higher proliferation potential as compared to those of the male sheep-derived MSCs. Female sheep MSCs also had rapid differentiation potential. The cryopreserved MSCs had morphological features comparable to that of the fresh cells. In conclusion, the tissue type and physiological status of donor animal may affect MSCs’ characteristics and should be taken into consideration while applying in clinical settings.

## 1. Introduction

Mesenchymal stem cells (MSCs) are increasingly being used in regenerative medicine due to their specialized properties of self-renewal, multiplication and differentiation, in addition to their ability to home-in and immune-modulate [1,2]. These cells are available in almost all the adult tissues and foetal membranes. Among various sources, mainly bone marrow (BM)- and adipose tissue (AD)-derived MSCs have been evaluated for in vivo applications, although foetal membrane-derived MSCs are being increasingly studied. There are various in vitro animal studies that have shown variability in the concentration, multiplication and differentiation properties of MSCs with respect to the tissue source [3,4,5,6,7,8,9,10], health status [11,12,13] and the physiological status of the donor animal [14,15], although with exceptions [16]. 

Some sources, such as bone chip, may harbour higher MSC concentration as compared to others, such as bone marrow [7]. The proliferation potential of MSCs also varies with respect to the tissue source as AD-MSCs tend to proliferate at a higher rate as compared to BM-MSCs and liver MSCs [5]. Even MSCs from a particular source may show differentiation potential more predisposed towards that particular lineage. MSCs from musculoskeletal sources such as bone marrow and synovial membrane have higher osteogenic differentiation potential as compared to those from adipose tissue [3,8]. This may further differ among the particular tissue sources as synovium MSCs may have higher chondrogenic potential than BM-MSCs [10]. 

Health status may also affect MSCs’ characteristics. In osteoporosis, goat BM-MSCs may have reduced proliferation and osteogenic differentiation potential [11]. Similarly, in equine metabolic syndrome (EMS), AD-MSCs are senescent and show lower proliferation potential [12]. In the case of bovine endometritis, endometrial MSCs show reduced proliferation and have little tendency towards adipogenic differentiation [13]. Anestral goat endometrial MSCs tend to show higher proliferation as compared to the cyclic endometrial MSCs [15]. MSCs’ proliferation potential reduces with the ageing, although a sheep study has failed to demonstrate such an observation [16]. All these studies show MSCs from particular sources at specific physiologies may have differential characteristics. Thus, such types of characteristics need detailed evaluation for effective utilization of MSCs in clinical settings. No literature on MSCs’ characteristics with respect to full term pregnancy in relation to the non-pregnant-derived tissue sources has been reported. Sheep are a suitable model animal for humans and previous studies using the species have been extrapolated for humans. The current study reports the effect of ambient temperature, tissue source and physiological status on characteristics of MSCs derived from sheep adipose tissue and bone marrow. 

## 2. Materials and Methods

### 2.1. Ethics Statement

The Institute Animal Ethics Committee of Sher-e-Kashmir University of Agricultural Sciences and Technology, Kashmir, India granted approval for this study vide order no. AU/FVSc/PS-57/15196. Sheep bone marrow and adipose tissue samples were harvested after obtaining written consent from the owners. All applicable national and/or institutional guidelines for the care and use of animals were followed.

### 2.2. Animals

The current study reports the characteristic features of mesenchymal stem cells (MSCs) derived from sheep (*Ovis aries*) bone marrow (BM-MSCs) and adipose tissue (AD-MSCs). The samples were collected from 19 mixed breed sheep (aged between 10 months to 2 years) that underwent caesarean section (Gender: Female; *n* = 9) and tube cystostomy (Gender: Male; *n* = 10), being restrained under the lumbo-sacral anaesthetic block. Initially, 5 samples (bone marrow (*n* = 3); adipose tissue (*n* = 2)) were transported and maintained for 30–45 min in the ambient temperature of < 10 °C (peak winter) until processing. Thereafter, the samples were transported and kept in a slightly improved temperature (ambient temperature >20 °C) till the processing. The samples were harvested in the small ruminant operation theatre of the Veterinary Clinical Complex, FVSc and AH. In female sheep, *n* = 7 samples of adipose tissue and *n* = 2 samples of bone marrow were harvested. In male sheep, *n* = 2 samples of adipose tissue and *n* = 8 samples of bone marrow were harvested. The culture and characterization work was conducted in the stem cell laboratory, Division of Veterinary Clinical Complex, FVSc and AH. 

### 2.3. Bone Marrow and Adipose Tssue Collection

BM was collected from iliac crest with a bone marrow biopsy needle (18 gauge) by an aseptic procedure. In short, the biopsy needle (stylet in place) was inserted into the bone and a syringe (10–20 mL), loaded with 2500 IU units of heparin (celparin, Celon Labs, Hyderabad, India) was attached to the needle. About 2.5 mL of bone marrow was aspirated into a hypodermic syringe and a similar procedure was followed on the other side to harvest equal volume. A total of 5.0 mL of BM collected was taken to the laboratory for processing. 

The adipose tissue (5.0 gm of omental fat) was collected from female sheep that underwent celiotomy (full-term caesarean section). The harvested tissue was maintained into Dulbecco’s phosphate buffer saline (DPBS) till the sample processing. 

### 2.4. Stem Cell Isolation and Culture Expansion

#### 2.4.1. Bone Marrow

BM samples were processed for isolation and culture expansion of MSCs as per the standard procedure [17]. BM samples were mixed with equal volume of Dulbecco’s phosphate buffered saline (DPBS) (Hyclone, GE Healthcare Life Sciences, Chicago, IL, USA) (1:1 *v*/*v*). The samples were passed through a 21-gauge intravenous catheter to disaggregate cell clumps and create a single cell suspension. Marrow samples with DPBS were loaded onto a half volume of Hisep LSM (HiMedia Laboratories, Mumbai, India) (1:0.5 *v*/*v*). The mono-nucleated cells collected from the interface were diluted with a double volume of DPBS (1:2 *v*/*v*) and collected by centrifugation at 300 gm for 30 min. After centrifugation, 3 mL of RBC lysis buffer (Himedia Laboratories) was added to the cell pellet for lysis of RBCs, if any, and again centrifuged at 200 gm for l0 min. The pellet obtained was washed with DPBS at the same speed of centrifugation. The cells were suspended in growth media (Dulbecco’s Modified Eagle’s Medium-low glucose (DMEM-LG) (Hyclone, GE Healthcare Life Sciences) +l0% foetal bovine serum (FBS) (VWR) and antibiotics (mixture of 100 units/mL of penicillin and 100 mg/mL of streptomycin (Himedia Laboratories). The cells were counted by Neubaeur’s counting chamber method (table), plated in T-25 culture flasks and maintained at 37 °C and 5% CO_2_ in a humidified atmosphere in a CO_2_ incubator (CellXpert, Eppendorf, Hamburg, Germany). The cells were plated at an average of 2.5 × 10^5^ in T-25 flasks. The non-adherent cells were removed after 3 days followed by media change (Table 1). Subsequently, growth medium (GM) was changed every 3 days. Upon 70–80% confluency (assessed by visual inspection under inverted microscope), the cells were passaged at lower densities into new culture flasks. The confluent flasks were trypsinized (0.075%) for about 3–5 min, followed by addition of GM to stop trypsin activity. The contents were collected in a centrifuge tube and centrifuged at 300 gm for 10 min. The supernatant was discarded and the pellet was re-suspended in 10 mL of GM. The single cell suspension was created after passing the cells through a 20-gauge needle three times. The cells were replated at the density mentioned earlier. Cultures were maintained at 37 °C and 5% CO_2_ in humidified conditions. For further cell passaging (up to passage 4) a similar procedure was followed. 

#### 2.4.2. Adipose Tissue

The isolation and culture expansion of AD-MSCs was performed as per the standard procedure [18] with slight modification. In brief, the collected fat was triturated and mixed in equal volume (1:1 *w*/*v*) of collagenase (1 mg/mL). The assembly was kept in a shaking incubator at 37 °C for 40 min. The enzymatic action was neutralized by adding equal volume of GM followed by centrifugation at 300 gm for 40 min. Half of the volume of the supernatant was removed followed by addition of GM, and the tubes were then centrifuged (200 gm for 20 min). The pellet obtained after discarding the supernatant was mixed with RBC lysis buffer (Himedia Laboratories, Mumbai, India) to lyse RBCs. The tubes were then again centrifuged at the same speed for 10 min. After complete RBC lysis, the clear white pellet was mixed in GM and implanted into the T-25 culture flasks at a density mentioned above and maintained at 37 °C and 5% CO_2_ in an incubator in a humidified environment. After obtaining the cell confluency of 70–80%, the cells were passaged as mentioned above up to passage 4.

### 2.5. Percent Adherent Cells (PAC) 

PAC from mononuclear cell (MNC) and stromal vascular fraction (SVF) were calculated after day 6 from initial cell seeding as detailed below:

PAC after initial seeding = *C*_2_/2*^h^*^1^^/^*^h^*^0^ × *C*_1_ × 100 (1)

where *h*_1_ = time in hours from initial seeding to calculation of total adherent cells (*C*_2_), and *h*_0_ = population doubling time of MSC.

### 2.6. Characterization of MSCs

The cells of two sources were characterized based on the morphology, surface marker expression and differentiation potential as recommended by the International Society for Cellular Therapy (ISCT) [19]. 

#### Morphology

Morphological characteristics of the MSCs were observed at different magnifications using an inverted microscope (LMI, Leeds, England, UK) at regular intervals.

### 2.7. Colony Forming Unit-f (CFU-f) 

The self-renewal property of the cells was observed by their colony forming capacity (CFU-f). The passage 3 cells were seeded at a low density and media were changed twice a week. To enumerate the colonies, PBS rinsed cells were fixed in 10% formalin at day 10. Aggregate of more than 50 cells stained with crystal violet (0.5%) (Himedia Laboratories, Mumbai, India) to lyse RBCs. The tubes were then again centrifuged at the same speed for 10 min. After) was considered as a colony, and total number of colonies was counted for the each cell line from two sources. 

### 2.8. Growth Kinetics and Population Doubling Time (PDT)

Growth kinetics of passage 3 MSCs was evaluated in culture media seeded in a 12-well plate at the rate of 10 × 10^3^ cells per well for each cell source. GM was changed twice a week. At every 48 h, two wells were harvested for each cell type and the cell number was counted using a haemocytometer. The growth curve was plotted for each type.

The cells (10 × 10^3^) were cultured in two 12-well culture plates. Two wells at 24 h intervals for 3 consecutive days were harvested and the cells were counted in each culture well using a haemocytometer. The population doubling time was calculated by:

PDT = Culture time (CT)/Cell doubling (CD)(2)

where CD = log (*C*_2_/*C*_1_)/log 2, *C*_2_ is harvested cell number, and *C*_1_ is initial cell number. Culture time is in hours.

### 2.9. Surface Marker Expression Analysis

The passage 3 MSCs from female and male sheep adipose tissue and bone marrow were analysed for their phenotypic marker expression (CD73, CD90, CD34, and CD45 markers) by reverse transcription polymerase chain reaction (RT-PCR).

#### Semi-Quantitative RT-PCR

Total RNA was extracted by RNeasy Micro Kit (Qiagen, Germantown, MD, USA) as per the manufacturer’s recommendations. Eluted total RNA samples were stored at −20 °C until use. Concentration and quality check were confirmed by Qubit^®^ 2.0 Fluorometer (Life Technologies, Carlsbad, CA, USA). The synthesis of first strand cDNA from RNA templates was conducted by following the manufacturer’s protocol (RevertAid First Strand cDNA Synthesis Kit, Thermo Scientific™). The product of the first strand cDNA synthesis was used directly in qPCR. A total of 2 µL of the first strand cDNA synthesis reaction mixture was used as template for subsequent PCR in the 25 µL reaction volume. Amplification reaction was set in a gradient thermal cycler (BioRad, Hercules, CA, USA). The results were analysed based on the presence or absence of specific amplification. Cycling conditions for different primers and their sequences are given in Table 1.

### 2.10. Tri-Lineage Differentiation

The tri-lineage differentiation was conducted on the MSCs (AD and BM) harvested from female sheep.

#### 2.10.1. Adipogenic Differentiation

Adipogenic differentiation of MSCs (10 × 10^3^ per 9.6 cm^2^) was evaluated using AdvanceSTEM Adipogenic differentiation kit (Hyclone, GE Healthcare Life Sciences, Bangalore, India). The cells from two sources were cultured for 3 weeks and evaluated through staining starting from day 10, as per the given instructions. Adipogenic differentiation was assessed by the presence of lipid droplets after staining with oil red O stain (Modified Promo Cell staining protocol). Expression analysis of PPARG with conventional RT-PCR (Table 1) was also made at day 15. As a negative control, an equal number of cells were maintained in the growth media for similar period.

#### 2.10.2. Chondrogenic Differentiation

Chondrogenic differentiation was evaluated by AdvanceSTEM Chondrogenic differentiation kit (Hyclone, GE Healthcare Life Sciences, Sciences, Bangalore, India) following the given instructions. MSCs (10 × 10^3^ per 9.6 cm^2^) from two cell types were cultured for 3 weeks and evaluated through staining starting from day 10. Differentiation was assessed by alcian blue staining. BGN expression with conventional RT-PCR (Table 1) was carried out on day 15. As a negative control, an equal number of cells were maintained in culture and supplemented with growth media for a similar period. 

#### 2.10.3. Osteogenic Differentiation

Osteogenic differentiation of each cell (10 × 10^3^ per 9.6 cm^2^) type was evaluated using an AdvanceSTEM Osteogenic differentiation kit (Hyclone, GE Healthcare Life Sciences, Sciences, Bangalore, India). Calcium deposition was evaluated by alizarin red staining (Modified Promo Cell staining protocol). As a negative control, an equal number of cells were maintained in the growth medium. The cells were cultured for 3 weeks with weekly medium changes and evaluated through staining starting from day 10. At day 15, expression analysis of BGLAP was also carried out with conventional RT-PCR (Table 1).

### 2.11. Cryopreservation and Post-Thaw Viability

Confluent cultures with an approximate cell number of 2.5 million cells of both sources (female sheep samples) were cryopreserved. The cells after trysinization and DPBS washing were resuspended into the 1 mL cryopreservation media (Hyclone, GE Healthcare Life Sciences) and kept in the 2 mL cryovials (GWare, Genetix, Asia Biotech Pvt Ltd., Hyderabad, India). The cryovials were kept in a 1 °C cooler and kept at −20 °C for 3–4 hrs, followed by overnight transfer to −80 °C; finally, the vials were kept in liquid nitrogen (LN2) over a 2-month period. Cryopreserved MSCs were revived by thawing them in a water bath at 37 °C. The cells were later poured into a 15 mL centrifuge tube containing 10 mL of GM. 

#### 2.11.1. Cell Viability 

The viability was measured by trypan blue staining as per the standard protocol [20]. In short, 10 μL of cell suspension was stained with equal volume of 0.4% trypan blue (1:1 *v*/*v*) (Himedia Laboratories, Mumbai, India) to lyse RBCs. The tubes were then again centrifuged at the same speed for 10 min. The stained cell suspension was loaded onto the Neubauer’s haemocytometer chamber and unstained cells were counted as live cells. The live percentage of cells was calculated as:

Post-thaw viability = live cell count/total cell count × 100(3)

#### 2.11.2. Cellular Expansion and CFU (f)

The cells of each source were culture expanded as per the protocol mentioned above. The cells were implanted in T-25 culture flasks (SPL Life Sciences, Pocheon-si, Korea). The CFU (f) was evaluated as described for fresh cells.

#### 2.11.3. Differentiation of Cells

The post-thaw cells from each source were put to differentiation for adipogenic, chondrogenic and osteogenic and evaluated as per the protocol described for fresh cells.

### 2.12. Statistical Analysis

The statistical analysis was conducted with IBM SPSS version 16. The independent t-test was applied to compare the means of two groups while for among the groups the statistical analysis was confirmed by the one-way ANOVA (analysis of variance) with Duncan’s post-hoc test. The level of significance was set at *p* < 0.05. The data are presented as mean ± standard error (SE).

## 3. Results

### 3.1. Bone Marrow and Adipose Tissue Collection

MSCs were isolated from bone marrow and adipose tissue of the sheep. Interestingly, we failed to isolate MSCs from samples (both the tissue types) under lower ambient temperature (<10 °C). All the samples at higher ambient temperature (>20 °C), however, were successfully processed and yielded MSCs. The overall success rate of MSC isolation from adipose tissue and bone marrow was 78% and 70%, respectively. 

### 3.2. Cell Population and Percent Cell Adherence (PAC)

**Figure 1:**
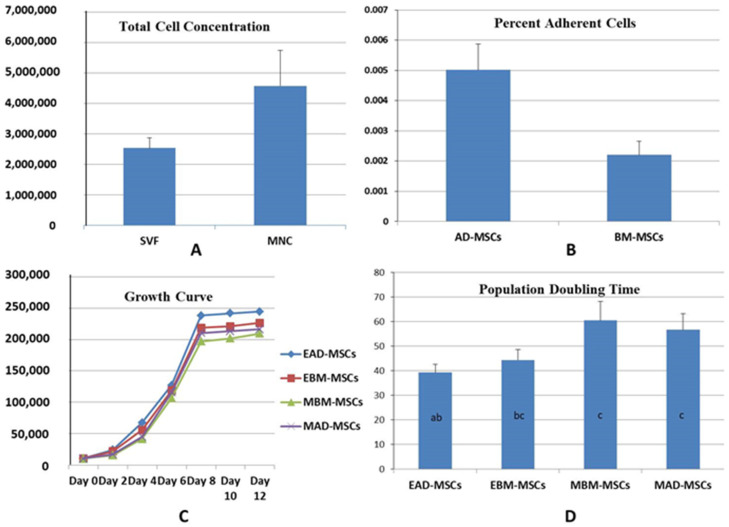
(**A**) The total initial cell concentration harvested from adipose tissue and the bone marrow. Values represented as mean ± SE. (**B**) Percent adherent cells (PAC) from stromal vascular fraction (SVF) and mononuclear cell (MNC) fraction. Values represented as mean ± SE. (**C**) Growth curve of the female adipose tissue MSCs (EAD-MSCs) and bone marrow MSCs (EBM-MSCs), and male sheep adipose tissue MSCs (MAD-MSCs) and bone marrow MSCs (MBM-MSCs). Female AD-MSCs and BM-MSCs had higher proliferation potential. (**D**) Population doubling time (PDT in hours) of the female adipose tissue MSCs (EAD-MSCs) and bone marrow MSCs (EBM-MSCs), and male sheep adipose tissue MSCs (MAD-MSCs) and bone marrow MSCs (MBM-MSCs). Female sheep AD-MSCs had a significantly (*p* < 0.05) lower PDT as compared to the other cell types. Bars with different letters differ significantly.

The initial cell count (stromal vascular fraction) for adipose tissue was lower as compared to bone marrow cell fraction (mononuclear cell fraction). The stromal vascular fraction (SVF) ranged from 0.97 million to 3.25 million with a mean ± SE of 2.53 × 10^6^ ± 3.43 per 5 gm of the tissue. Mononuclear cell fraction (MNCs) ranged from 2.05 million to 6.5 million with a mean ± SE value of 4.57 × 10^6^ ± 6.2 per 5 mL of bone marrow (Figure 1A). The initial cell count and average PAC were higher in MNCs and SVF, respectively, but statistically non-significant (*p* > 0.05) (Figure 1B).

### 3.3. Growth Kinetics and PDT

The cells were maintained in GM up to passage 4. The growth characteristics of sheep BM-MSCs and AD-MSCs had shown a typical growth curve encompassing lag, log and plateau phases. Female sheep AD-MSCs had a lag phase of 24–36 h, while in female sheep BM-MSCs and male AD-MSCs and BM-MSCs, a lag phase of 1–2 days was seen. A log phase of 7–8 days followed by plateau up to day 12 was seen. Thereafter a declined growth rate was seen in all these cells (Figure 1C). 

The PDT for female sheep AD-MSCs and BM-MSCs ranged from 29.41 h to 51.61 h (mean 39.26 h) and 32.74 h to 59.70 h (mean: 44.35 h), respectively. The PDT for male sheep-derived BM-MSCs and AD-MSCs ranged from 39.05 h to 86.96 h (mean 60.51 h) and 38.30 h to 74.53 h (mean: 56.73 h) (Figure 1D). Female sheep AD-MSCs had a significantly (*p* < 0.05) lower PDT as compared to the male sheep AD-MSCs and BM-MSCs, while female sheep BM-MSCs had lower PDT but statistically non-significant (*p* > 0.05) as compared to male sheep tissue-derived cells. 

### 3.4. Cell Morphology

**Figure 2:**
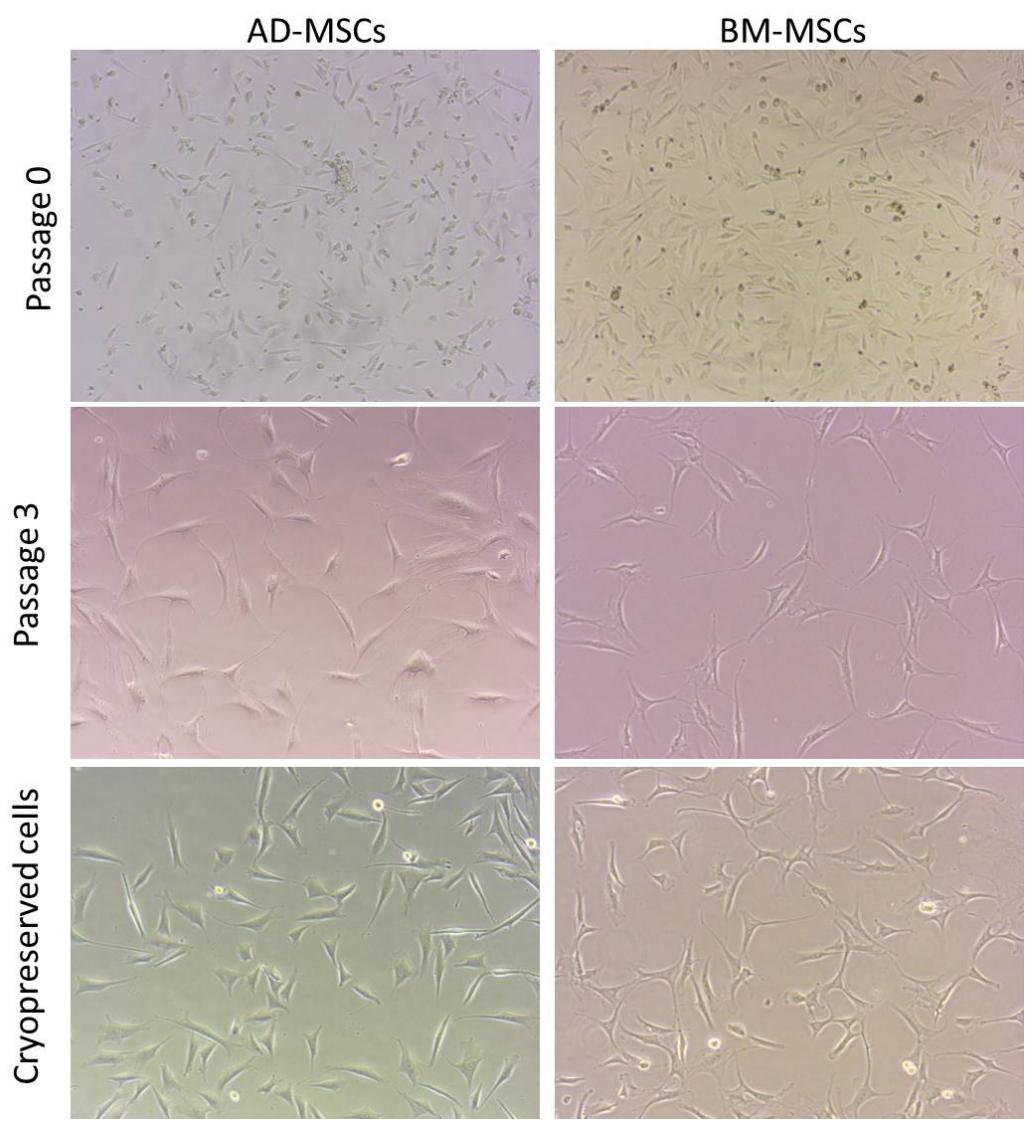
Plastic adherent mesenchymal stem cells (MSCs) of adipose tissue and bone marrow-derived tissues. The cells at passage 0 had varied morphology while cells at later passage (P3) had typical fibroblast morphology. P0: cells at passage 0; P3: cells at passage 3; Cryopreservation: cell confluency (P4) after cryopreservation (Magnification: 20×).

The adherent cells initially (P0–P1) were of mixed shapes ranging from polygonal, rounded to spindle shaped fibroblast-like cells. Subsequently, in advanced passage (P2–P4) the cells had typical uniform spindle shaped fibroblast-like morphology (Figure 2). 

### 3.5. Colony Forming Unit (Fibroblasts) Assay

**Figure 3:**
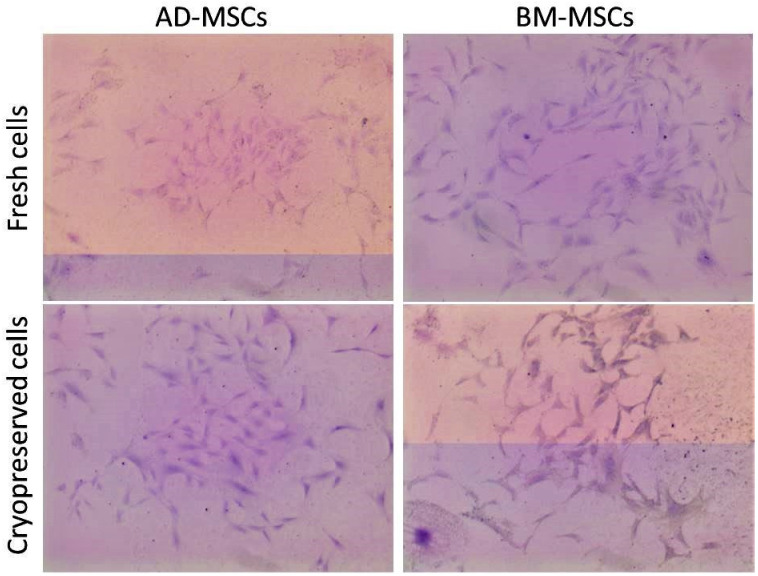
Colony forming unit (fibroblast) formation of MSCs from adipose tissue (AD-MSCs) and bone marrow (BM-MSCs). The passage 3 cells seeded at a low density were counted for aggregates of more than 50 cells stained with crystal violet (0.5%) (Magnification: 4×).

Female sheep AD-MSCs had a higher clonogenic potential (48 clones in all the fields) followed in decreasing order by female BM-MSCs (44 clones in all the fields), male sheep-derived AD-MSCs (36 clones in all the fields) and BM-MSCs (27 clones in all the fields) (Figure 3).

### 3.6. Phenotypic Gene Expression

**Figure 4:**
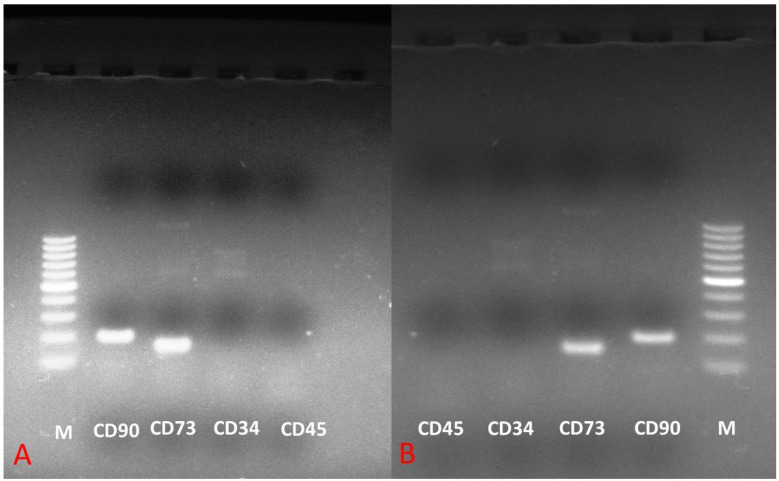
The expression analysis of genes of adipose tissue MSCs (**A**) and bone marrow MSCs (**B**) for different phenotypic markers. M: 50 bp Ladder. The phenotypic surface markers expressed are CD73 and CD90, while haematopoietic markers unexpressed were CD34 and CD45.

MSCs from two cell sources evaluated for surface markers (CD73 and CD90) and haematopoietic markers (CD34 and CD45) revealed positive surface marker expression while expression for haematopoietic markers was lacking (Figure 4). 

### 3.7. Tri-Lineage Differentiation

**Figure 5:**
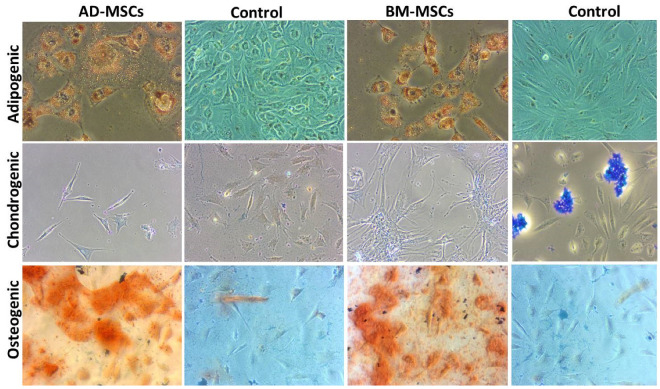
Images demonstrate that adipose tissue MSCs and bone marrow MSCs differentiated into adipogenic, chondrogenic and osteogenic lineages. The confirmation was conducted with specific staining. Adipogenic differentiation confirmed by oil red O staining (fat droplets stain red); chondrogenic differentiation confirmed by alcian blue (proteoglycans stain bluish); osteogenic differentiation confirmed by alizarin red (calcium crystals stain red). The control samples (MSCs in growth media) are along the specific differentiated cell line (Magnification: 20×).

**Figure 6:**
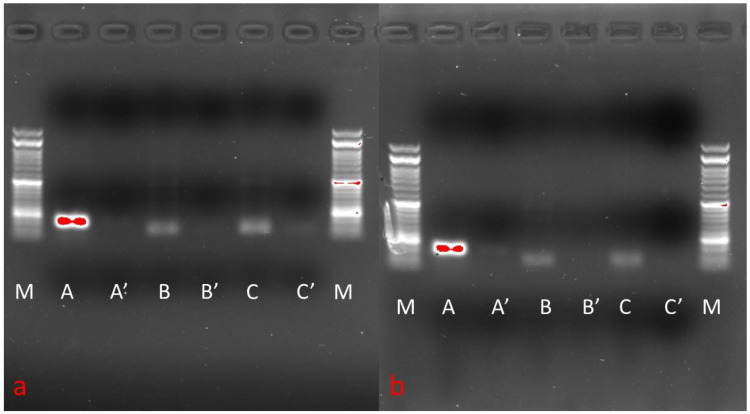
The expression analysis of genes of adipose tissue MSCs (**a**) and bone marrow MSCs (**b**) for different differentiation markers. M: 50 bp Ladder. The differentiation markers expressed are PPARG (Adipogenic: A differentiated; A′ undifferentiated), BGN (Chondrogenic: B differentiated; B′ undifferentiated) and BGLAP (Osteogenic: C differentiated; C′ undifferentiated).

MSCs from two sources (AD and BM) were subjected to tri-lineage differentiation post-establishment of the monolayer. MSCs from these two cell sources were comparable in their ability to differentiate into the adipogenic, chondrogenic and osteogenic lineages, as demonstrated by the special staining techniques. MSCs from both the sources could differentiate into adipogenic and chondrogenic as early as 10 days and effective osteogenic differentiation was achieved by day 15. MSCs could maintain morphology in chondrogenic differentiation until 10 days, while in adipogenic differentiation cellular morphology was changed and cells started detaching as early as 6–8 days. Osteogenic characteristics started appearing post 10 days and better at day 15. The staining of chondrogenic, osteogenic and adipogenic-differentiated MSCs was characteristic for proteoglycans, calcium crystals and oil droplets, respectively (Figure 5). The semi-quantitative RT-PCR-based gene expression analysis showed higher expression of PPARG, BGN and BGLAP corresponding to the adipogenic, chondrogenic and osteogenic differentiation, respectively, in concerned differentiation cocktails (Figure 6). 

### 3.8. MSCs Cryopreservation and Post-Thaw Characteristics

The cryopreserved female sheep-harvested AD-MSCs and BM-MSCs could successfully revive with a viability percentage of 90% and 89.6%, respectively. The cells could be successfully re-cultured. The cellular morphology appeared fibroblast-like. The cryopreserved cells successfully achieved >80% confluency within 8–10 days (Figure 2). The CFU (f) was comparable to that of the fresh cells although a bit lower (44 colonies in AD-MSCs and 42 colonies in BM-MSCs) (Figure 3). These cells also had successful and similar differentiation properties to those of the fresh cells. 

## 4. Discussion

MSCs are being increasingly utilized in regenerative medicine with a positive in vivo healing response, although currently no definitive use in clinical settings has been made. The main limitation that restricts their definitive utilization is the lack of understanding of their cellular physiological processes [2,23]. MSCs lack uniform properties across donor tissues and also with respect to the physiological status of the donor. For reliable and effective clinical utilization of MSCs, it is imperative to study their properties with respect to the tissue source and physiological status of the donor. The current study demonstrates MSCs’ properties harvested from adipose tissue and bone marrow at full term pregnancy and from male donors suffering with urolithiasis.

In the current study, unsuccessful attempts were made to harvest MSCs in peak winter. The transportation and maintenance of the samples at lower temperature for around 30–45 min might have affected the tissue samples. Some of the studies have been able to demonstrate isolation of MSCs from cryopreserved foetal membranes and umbilical cord blood, although with limited success [24,25,26]. The success rate of MSCs’ isolation from umbilical cord blood had been <60% and a delay in achieving the culture expansion has been demonstrated [24,25]. Furthermore, MSCs’ isolation from foetal membranes has been achieved through the explant method [26]. In the current study, the direct exposure to cold might had led to cold shock to the cells; moreover, the adipose tissue under cold environment undergoes hardening, which complicates its enzymatic digestion. The adipose tissue had been processed through an enzymatic method, which could have further stressed the cells. 

As the tissue samples contain a miniscule number of MSCs, it becomes imperative to culture expand and characterize them before in vivo application. There are variable cell concentrations in tissue sources, and they also tend to vary as per the physiological status of the donor. MNC fraction isolated from bone marrow samples had higher cell concentration as compared to the SVF derived from adipose tissue. The higher cell concentration in bone marrow as compared to that of adipose tissue in the current study is similar to that reported for equines. However, comparably less initial cell concentration in both bone marrow as well as adipose tissue was particular to our study as compared to that demonstrated for equine tissues [27,28]. The higher percent adherent cells in SVF as compared to MNC fraction in our study suggests that a higher MSCs fraction may be available in adipose tissue than bone marrow. The limited cell concentration, however, can be compensated for by harvesting more donor tissue for desired therapeutic concentrations. 

Although AD-MSCs had shorter PDT as compared to the BM-MSCs, considerable differences with respect to PDT were evident in the samples harvested from full term parturient female sheep as compared to those MSCs harvested from male sheep. One of the previous sheep studies had demonstrated lower proliferation potential of AD-MSCs as compared to the BM-MSCs [5]. The possible reasons behind such a significantly higher proliferation rate and higher cellular concentrations of MSCs from full term parturient female sheep may be due to the higher systemic cortisol and oestrogen concentrations at parturition [29,30,31]. The higher oestrogen concentration at term might favour epithelial-to-mesenchymal transition [31]. However, a delicate balance of such hormones is required as higher cortisol concentration can be detrimental to cells [30] and in absence of progesterone, estrogenic effect may not be obvious [31]. As urolithiasis (urinary obstruction) is an acute condition, the adverse effect on the cell count or their growth is remote. It is also worth mentioning that although the effect of the above-mentioned hormones remains transient, in current study the cells showed higher proliferation for advanced passages and, thus, further studies are desired. 

The cells from two sources effectively followed the standards of the ISCT [19]. Both these cell types were plastic adherent and appeared fibroblast-like, although at early passage the cells exhibited diverse morphology as has also been reported in other studies [32,33]. The two cell types positively expressed genes for some surface markers while simultaneously lacking expression for haematopoietic marker expression. The two cell types effectively underwent tri-lineage differentiation. These cells showed chondrogenic and osteogenic differentiation as early as 10 days, while most of the studies have reported a 2–3-week time period for cellular differentiation. The possible reason for early chondrogenic and adipogenic differentiation of MSCs may be due to the estrogenic effect [31]. However, contrary to the oestrogen effect reported for MSCs’ osteogenic differentiation [31], we could achieve osteogenic differentiation by day 15 and, thus, further insights are desired. On gene expression analysis, adipogenic (PPARG), chondrogenic (BGN) and osteogenic (BGLAP)-specific genes were upregulated in differentiated cells. These findings have also been reported by others [21,22]. However, one study failed to show higher BGN expression in the chondrogenic differentiation cocktail [21], contrary to another study [22]. This could possibly be due to the differences in the cells based on the source and the physiological status of the donor. Comparative phenotypic and differentiation marker expression potential of MSCs from parturient female and male sheep may be studied for better understanding.

The cryopreserved cells from two sources had a comparable viability. MSCs’ viability from two sources was comparably better than that reported for goat foetal membrane-derived MSCs [20] but slightly lower than that reported for sheep dermis-derived MSCs [34]. The cellular viability is affected by the donor tissue type and cell freeze-thaw procedure and needs further advancements to enable the harvesting of an acceptable percentage of cells. The cellular features such as morphology, growth and differentiation were comparable to those of the fresh cells, in agreement with results reported for sheep BM-MSCs [16] and foetal membrane MSCs [20]. 

## 5. Conclusions

Tissue samples transported under cold ambient temperature may not be suitable to isolate MSCs, irrespective of the donor tissue type. The tissues harvested from full term pregnant female sheep may yield MSCs with improved proliferation and differentiation potential. The cryopreserved cells tend to show similar cellular features to those of fresh cells and may be a suitable source for their ready-to-use application(s). 

## Figures and Tables

**Table 1:** Primer sequences of phenotypic markers (F: Forward and R: Reverse) and the length of the amplicon in base pairs (bp).

Sl. No	Gene	Primer Sequence	Annealing Temperature	Amplicon Size (bp)	References
1.	CD73	F-CTGAGACACCCGGATGAGAT R-ACTGGACCAGGTCAAAGGTG	55 °C	160	[20]
2.	CD90	F-GTGAACCAGAGCCTTCGTCT R-GGTGGTGAAGTTGGACAGGT	55 °C	201	
3.	CD34	F-TGGGCATCGAGGACATCTCT R-GATCAAGATGGCCAGCAGGAT	60 °C	107	[21]
4.	CD45	F-CCTGGACACCACCTCAAAGCT R-TCCGTCCTGGGTTTTATCCTG	60 °C	101	
5.	BGLAP	F-CCCAGGAGGGAGGTGTGTG R-CTAGACCGGGCCGTAGAAGC	58 °C	99	
6.	BGN	F-AACATGAACTGCATTGAGATGGG R-GCGAAGGTAGTTGAGCTTCAGG	58 °C	93	
7.	PPARG	F-ACGGGAAAGACGACAGACA R-AAACTGACACCCCTGGAAGATG	56 °C	150	[22]

## Data Availability

Available data are presented in the manuscript.

## Abbreviations

MSCs: Mesenchymal stem cells; BM: Bone marrow; AD: Adipose tissue; SVM: Stromal vascular fraction; MNC: Mononuclear cell; PAC: Percent adherent cells; ISCT: International Society for Cellular Therapy; DPBS: Dulbecco’s phosphate buffered saline; DMEM-LG: Eagle’s Medium-low glucose; FBS: Foetal bovine serum; PDT: Population doubling time; RT-PCR: Reverse transcription polymerase chain reaction; CFU: Colony forming unit; ANOVA: Analysis of variance; SE: Standard error.
